# Introduction of a PRRSV-1 strain of increased virulence in a pig production structure in Spain: virus evolution and impact on production

**DOI:** 10.1186/s40813-022-00298-3

**Published:** 2023-01-03

**Authors:** Gerard E. Martín-Valls, Martí Cortey, Alberto Allepuz, Francesc Illas, Montserrat Tello, Enric Mateu

**Affiliations:** 1 Dept. Sanitat i Anatomia Animals, Facultat de Veterinària, Universitat Autònoma de Barcelona, Travessera dels Turons s/n, Campus UAB, 08193 Cerdanyola del Vallès, Spain; 2 Grup Batallé, Avinguda dels Segadors s/n, 17421 Riudarenes, Spain

## Abstract

**Background:**

A strain *of Porcine reproductive and respiratory syndrome virus* (PRRSV), showing characteristics of enhanced virulence, affected a pyramidal production system from Spain with 7600 sows in 4 genetic nuclei and 13,000 sows in multipliers. Different PRRSV strains circulating in this production system from December 2020 to October 2021 were detected and sequenced. The spread of each isolate was examined and their impact on health and production in three of the affected farms was evaluated.

**Results:**

The newly emerged PRRSV isolate with enhanced virulence entered the system before the onset of the study (January 2020) and afterwards four significantly different clades were detected during the study period in different farms, probably because of independent introduction events. The diversification of the enhanced virulence strain was higher for those clades (substitution rates up to 1.1% nucleotides/year) compared to other PRRSV strains present in the production system (up to 0.17%), suggesting a faster spread and adaptation. The impact of the infection in the first affected farm was dramatic, with an average abortion rate above 27% during 17 weeks before returning to the baseline production. Fertile sow mortality reached 6.5% for 39 weeks. In two farms infected later by other clades of this enhanced virulence strain, the impact was less acute; despite the fact that for parameters such as the proportion of stillbirths or mummies, more than 10 months were needed to recover pre-outbreak values. In the examined nurseries, mortalities reached peaks between 28 and 50% and several months were needed to return to normality.

**Conclusion:**

Introduction of a PRRSV strain of enhanced virulence in a production system where several farms were previously positive for other PRRSV strains, resulted in a fast spread such as would be observed in naïve farms. The productive and health impact was very high taking several months to return to normality.

**Supplementary Information:**

The online version contains supplementary material available at 10.1186/s40813-022-00298-3.

## Background

Porcine reproductive and respiratory syndrome (PRRS) is one of the most economically important diseases of pig industry. According to Holtkamp et al. [10] the disease cost about U$ 664 million annually to the American industry, of which weaners and growers accounted for 55% of the total cost. In Europe, Nieuwenhuis et al. [20] estimated an average loss of € 126 per sow (€ 59–379) during an outbreak period. More recently, Nathues et al. [19] estimated a cost of 126.79 €/sow/year in a farm with slight reproductive problems, and 3.77 €/fattener in a farm with slight respiratory disease.

Losses caused by PRRS result from reproductive disorders, such as abortions and stillbirths, but also from an increased proportion of weak-born piglets and increased mortality in new-borns [2, 6, 25]. Besides, PRRSV is one major component of the porcine respiratory disease complex, and the presence of PRRSV circulating in the nurseries commonly leads to increased mortality when combined with other bacterial or viral agents [4, 9, 28]. In many affected nurseries, the presence of PRRSV results in increased medication costs as well [27]. Indirect costs, arising from the distortion caused by the disease on the normal production flow together with control costs, are also important.

PRRS is caused by *Betaarterivirus suid* 1 and 2 viruses, namely PRRS virus (PRRSV) 1 and 2. One of the important features of PRRSV is its high genetic and antigenic diversity, which is constantly evolving [18]. PRRSV-1, the predominant species in Europe, is subclassified in 4 subtypes, each one of which include several clades [22]. In Western Europe, subtype 1 (PRRSV-1.1) is predominant. This genetic diversity also translates to a variable virulence. In general, PRRSV-1 was considered less virulent than PRRSV-2 [17], a notion reinforced by the emergence of an extremely virulent strain in China some years ago [26]. However, the discovery of strains like Lena in Belarus (PRRSV-1.3) [11], or PR40 (PRRSV-1.1) in Italy [5], demonstrated the existence of highly virulent PRRSV-1 strains.

Starting in 2020, severe PRRSV outbreaks characterized by high abortion rates and increased mortality rates in weaners, were increasingly reported in North-eastern Spain (NE Spain) [16]. Isolation and sequencing of the virus in several outbreaks [15] showed that they were caused by a mosaic PRRSV strain, derived from the PR40 strain reported by Canelli et al. [5], after drifting and undergoing several recombination events with other PRRSV-1.1 isolates.

The aim of the present study was to assess the evolution of this PRRSV strain with enhanced virulence after its introduction in a production system. The productive impact of the strain in selected breeding sites and nurseries was also examined.

## Results

### Emergence of the highly virulent PRRSV isolate in the production system

**Fig. 1:**
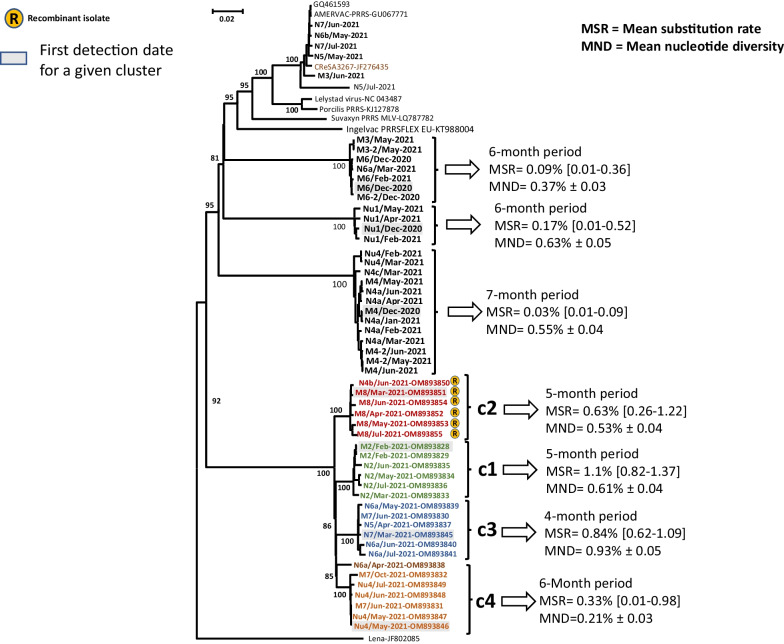
Maximum likelihood phylogenetic tree build up using whole genome sequences, a general time reversible model with 1,000 iterations and pairwise deletion. Numbers along the branches represent the confidence probability of the corresponding clade

Four different clades of the newly emerged highly virulent PRRSV were detected (c1-c4) (Fig. 1). The first detection (c1) of the new highly virulent strain occurred before the start of the present study, in farm M2 in January 2020; no other outbreaks occurred until the beginning of 2021. In March 2021, two new outbreaks of the highly virulent strain were reported. The first occurred in the farrow-to-finish farm M8 (c2). This same virus was later found in nursery N4b (June 2021). The second detection (c3), appeared in nursery N7 in March 2021, and shortly thereafter (April 2021 onwards) in nurseries N5 and N6a, and in June 2021 in farm M7. The fourth clade (c4) appeared in May 2021 in the nucleus Nu4 and then spread to M7 in June that year. The source of infection for the original outbreak of each clade could not be identified. Subsequent outbreaks in other farms were likely the result of the movement of animals to nurseries. All other farms remained with its previous PRRSV status as of December 2020. Additional file 1 shows the chronology of positive PRRSV detections in the system.

### Evolution of the newly emerged PRRSV isolate

Next, we analyzed the diversification of the newly emerged highly virulent PRRSV isolate in comparison to the isolates previously present in the system (Fig. 1). The substitution rates (expressed as percentage of yearly changes in the whole genome) for the newly emerged strain ranged from 0.33 to 1.11%, compared to 0.03%-0.17% for the previous PRRSV strains present in the system. In contrast, the mean nucleotide diversity was similar between the newly emerged and the previous strains (0.21–0.93% versus 0.37–0.63% for the pre-existing ones). In general, the highest rates within the genome were observed for segments nsp2, ORF3, ORF4, ORF5, with nsp9 and nsp10 showing the lowest. Of note, the pattern was not different when newly emerged or previous strains were compared. The highly virulent isolate was identified as a mosaic, with a backbone derived from a strain related to the isolate PR40 [5] that incorporated four recombinant segments. During the study, an additional recombination event involving nsp9 was detected in all isolates of c2. A new recombinant isolate was detected in nursery N5 in July 2021. In this case, the virus contained the backbone of a vaccine strain (UNISTRAIN®), with a recombinant segment in ORF1b (region encoding nsp9) derived from the highly virulent PRRSV isolate. Additional file 2 shows the recombination events found for the newly emerged strain.

### Impact of the infection by the newly emerged PRRSV virus on PRRSV vaccinated farms

To analyze the impact of the infection in different farms, productive data of farms M2 (c1), M7 (c3 and c4) and Nu4 (c4) were examined. These farms were infected at different moments of the dissemination of the new highly virulent strain (January 2020 to April 2021). M2 had been detected as positive stable for a Spanish clade of PRRSV-1 before January 2020, when reproductive problems started again; in that case caused by the highly virulent PRRSV strain. M7 was PRRSV positive stable until December 2020, when a reproductive outbreak characterized by abortions started. Nu4, that was positive for a Spanish clade of PRRSV1, suffered a reproductive outbreak starting April 2021 (confirmed in May 2021).

**Fig. 2:**
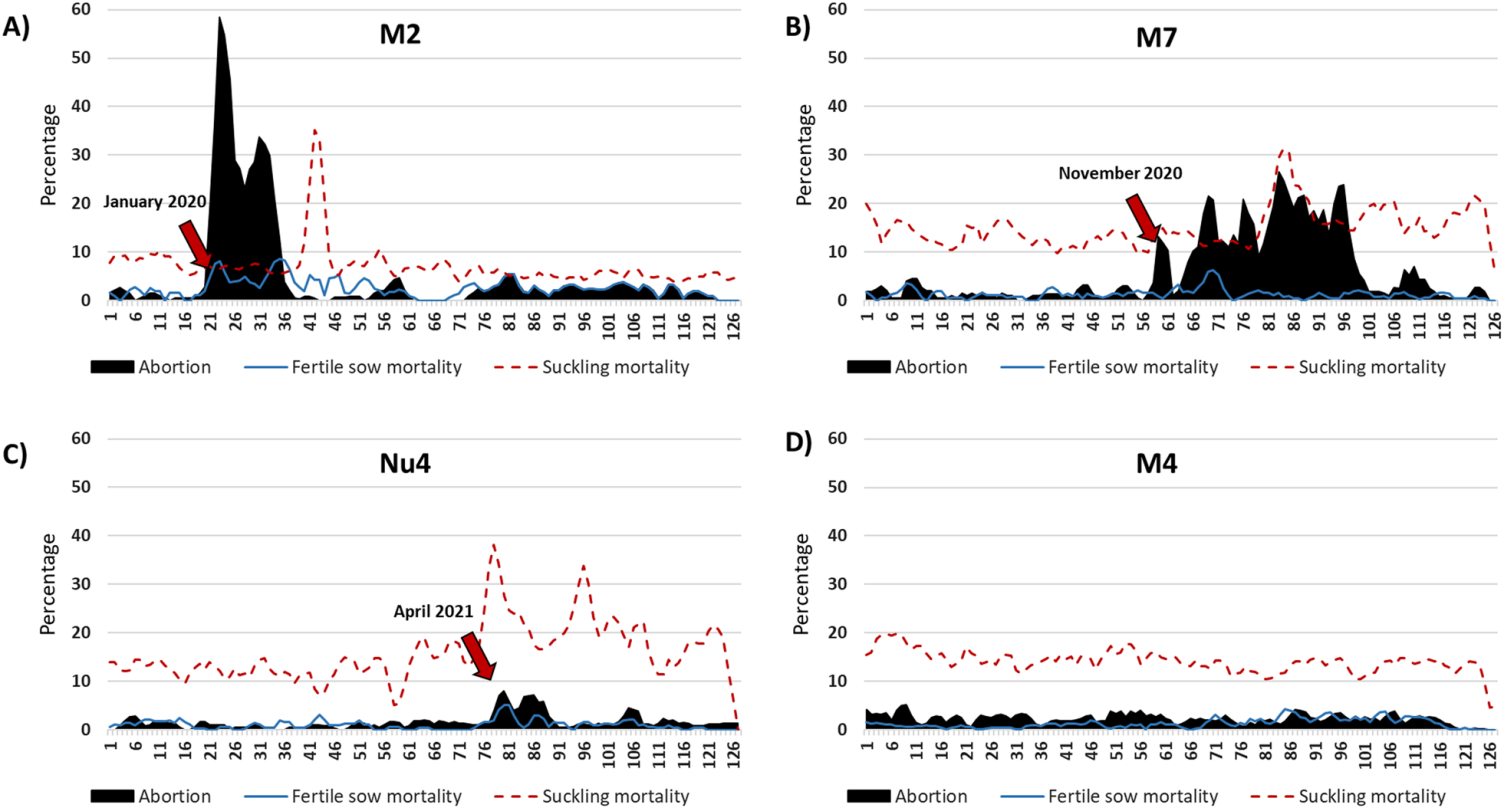
Impact of an increased virulence PRRSV isolate compared to a strain of moderate virulence. The graphs depict the temporal evolution of the abortion rate, fertile sow mortality and suckling piglet mortality starting on the 31st week of 2019 until the beginning of 2022. **A**–**C** farms (M2, Nu4 and M7) that suffered outbreaks caused by the increased virulence PRRSV strain. **D** A farm (M4) that was endemic instable for a Spanish PRRSV isolate of moderate virulence. The red arrows indicate the starting of the outbreak for each A, B and C farms, farm D had been recurrently positive since before 2019

**Fig. 3:**
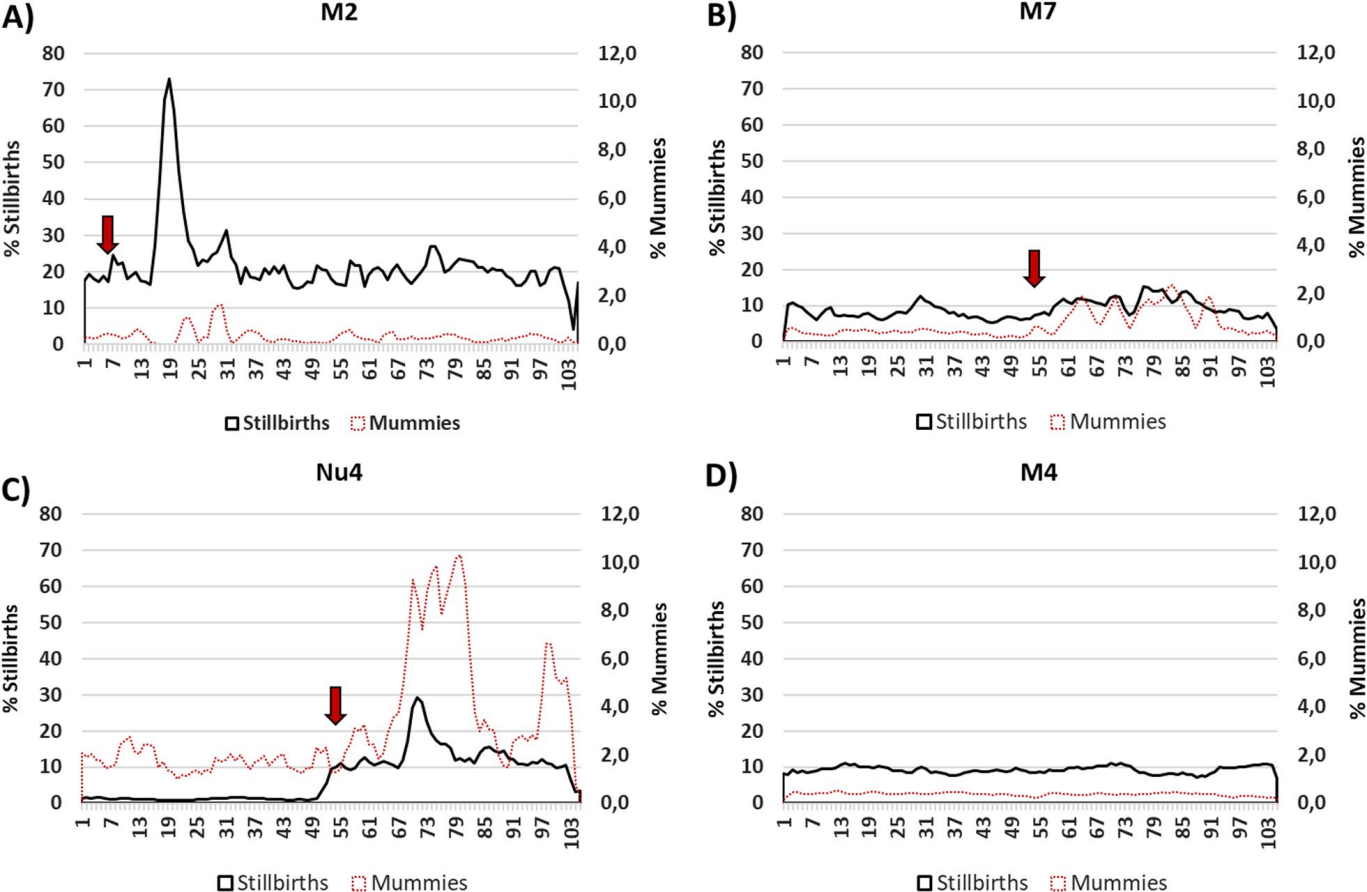
Temporal evolution of stillbirths and mummies in farms suffering PRRSV outbreaks of increased virulence compared to a farm infected by a strain of moderate virulence. The graphs show the percentages of stillbirths and mummies during 2020 and 2021 in three farms:M2 (**A**), M7 (**B**) and Nu4 (**C**) affected by the PRRSV strain of increased virulence compared to a PRRSV endemic farm (M4, **D**) where a moderate virulence isolate was circulating since before 2019. The red arrow shows the onset of the reproductive PRRS outbreak in each farm

Table 1 and Figs. 2 and 3 summarize the impact of the infection on productive parameters of the examined farms. Additional file 3 shows the impact of the disease on the actual number of weaned piglets versus the expected values. Additional file 4 shows the excess on mortality in nurseries. In M2, probably one of the first farms infected by the emerging highly virulent strain in Spain, the impact of the infection was devastating. For 13 consecutive weeks the abortion rates surpassed 25%, reaching 71% some weeks; the return to baseline production required 17 weeks. In this farm, the death rate of fertile sows was affected for 39 weeks after the onset of the outbreak, with a weekly average of 6.5%. During the outbreak, mortality of suckling piglets was little affected because summing up abortions, stillbirths and mummies some weeks the count of piglets born alive was close to zero.

**Table 1:** Summary of several productive parametres before and during PRRS outbreaks caused by the strain of enhanced virulence

	Before the outbreak	During the outbreak	Weeks to baseline
	**FARM M2**		
Abortion rate	Average: 1.0% Median: 0.7%	Average: 28.7% (+ 27.7)**** Median: 29.2% (+ 28.5) Peak: 71.0%	17
Stillbirths	Average: 6.8% Median: 5.7%	Average: 9.4% (+ 2.6)* Median: 8.4% (+ 2.7) Peak: 27.0%	17
Mummies	Average: 0.6% Median: 0.7%	Average: 1.8% (+ 1.2) (n.s) Median: 1.7% (+ 1.0) Peak: 9.6%	16
Fertile sow mortality rate	Average: 1.0% Median: 0.8%	Average: 6.5% ( +)*** Median: 4.8% ( +) Peak: 25.0%	39
Suckling piglet mortality	Average: 10.9% Median: 7.9%	Average: 7.3% (-2.6) (n.s.) Median: 6.3% (-1.6) Peak: 10.6%	0
	**FARM M7**		
Abortion rate	Average: 1.5% Median: 1.4%	Average: 11.1% (+ 9.6)**** Median: 9.5% (+ 8.1) Peak: 35.4%	42
Stillbirths	Average: 8.3% Median: 8.0%	Average: 13.0% (+ 4.7)* Median: 13.2% (+ 5.2) Peak: 16.7%	37
Mummies	Average: 2.9% Median: 2.8%	Average: 6.8% (+ 3.9)** Median: 4.6% (+ 1.8) Peak: 28.5%	40
Fertile sow mortality rate	Average: 1.2% Median: 1.3%	Average: 3.7% (+ 2.5) *p* = 0.1 Median: 3.2% (+ 1.9) Peak: 7.9%	11
Suckling piglet mortality	Average: 13.5% Median: 12.8%	Average:15.0% (+ 1.5)*** Median: 16.7% (+ 3.9) Peak: 31.3%	Not yet reached after 42 weeks
	**FARM Nu4**		
Abortion rate	Average: 0.7% Median: 0.7%	Average: 5.9% (+ 5.2)**** Median: 5.6% (+ 4.9) Peak: 10.4%	12
Stillbirths	Average: 1.6% Median: 1.2%	Average: 10.7% (+ 4.7)**** Median: 10.8% (+ 5.2) Peak: 37.1%	Not reached yet after 39 weeks
Mummies	Average: 1.7% Median: 1.7%	Average: 2.6% (+ 0.9)** Median: 2.4% (+ 0.7) Peak: 11.5%	Not reached yet after 39 weeks
Fertile sow mortality rate	Average: 0.7% Median: 0.7%	Average: 2.5% (+ 1.8)** Median: 2.1% (+ 1.4) Peak: 5.2%	39
Suckling piglet mortality	Average: 12.2% Median: 12.6%	Average: 16.1% (+ 3.9)**** Median: 15.6% (+ 3.0) Peak: 38.3%	Not reached yet after 39 weeks

The table shows the main indicators for the farrowing units before and during the outbreak and the time needed to recover the baseline values. Statistical significance (Mann–Whitney): *p < 0.05; **p < 0.01; ***p < 0.001; ****p < 0.0001; n.s. = non significant

In M7, infected by the strain of increased virulence 11 months later (December 2020), the impact was more persistent. Thus, abortion rates reached on average only 11%, but 42 weeks were required to return to the baseline. Interestingly, in M7 the proportion of mummies was very high (6.8%) for 40 weeks. In this case, mortality of sows was lower and non-significantly different from the rates recorded before the outbreak. Mortality of suckling piglets was not restored to normal values before the 43^rd^ week after the onset of the outbreak.

In Nu4, the abortion rate increased less than in the other two farms (5.9% on average) and the return to baseline only required 12 weeks. In contrast, the proportion of stillbirths and mummies remained elevated for more than 39 weeks. The sow death rate although lower (2.5%) when compared to M2, remained elevated for 39 weeks in comparison to the pre-outbreak values. Suckling piglet mortality remained elevated for more than 39 weeks.

For comparison purposes, for breeding farm M4, that had been infected endemically with PRRSV since before 2019, the average figures for the period 2019–2020–2021 were: abortion rate: 2.3% ± 1.03%; mortality rate of fertile sows: 1.3 ± 1.2%; stillbirths: 9.2% ± 2.0%; mummies: 0.4 ± 0.09/litter, pre-weaning mortality: 14.3% ± 2.6%.

**Fig. 4:**
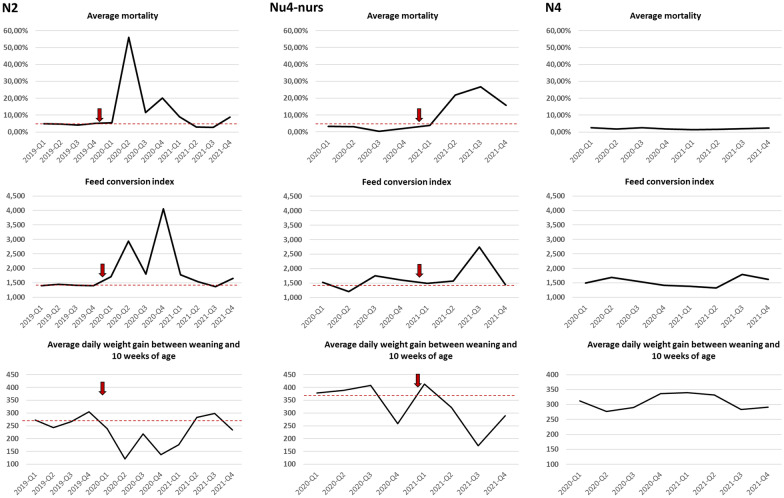
Evolution of mortality, feed conversion index and average daily weight gain in nurseries N2 and Nu4-nurs, after being infected with a highly virulent PRRSV-1 isolate, compared to nursery N4 that received piglets from a farm endemically infected with a PRRSV-1 strain of moderate virulence. The dotted read line indicates the median of each parametre before the outbreak. Data are shown per trimesters (Q1–Q4 each year)

Nurseries N2 and Nu4-nur that were filled with animals from M7 and Nu4 were also followed (Fig. 4). In both cases, the impact on mortality from weaning to the end of the nursery phase was evident, reaching peaks of almost 60% in N2. Return to normality required several months. The feed conversion index and the average daily weight gain were severely affected as well.

## Discussion

PRRS is one of the costliest diseases of pigs. For many years, it was considered that the species PRRSV-2 included strains of higher virulence compared with the species PRRSV-1. However, in the last years, the description of isolates such as Lena (PRRSV-1.3) or PR40 (PRRSV-1.1) [5, 11] suggest that PRRSV-1 also include strains of elevated virulence.

During the first half of 2020, a new highly virulent PRRSV strain emerged in Spain [15]. That strain was shown to derive from a previously reported strain circulating in Italy [5]. However, the strain circulating in Spain have undergone several recombination events with other PRRSV-1 isolates. Some of these recombination events probably took place locally in Spain, as identified in the analysis of the available sequences. Whether these recombination events resulted in enhanced virulence is not known but, in our opinion, this is a likely hypothesis, since all full genome sequences of this highly virulent isolate known to us harbor these recombining segments.

It is difficult to determine the precise source of introduction of the new PRRSV strain in the studied farms. In the case of farm M2, the first case in the studied production system and one of the first reported cases in Spain, no animals were introduced from external sources and introduction by people or fomites was unlikely given the biosecurity characteristics of the herd. However, M2 was located in a flat area about 800 m from a fattening unit belonging to a different company that frequently imported fatteners from other locations and that suffered a sudden increase of mortality just before the outbreak in M2, supposedly caused by PRRSV. In this case, airborne transmission from the fattening unit to M2 cannot be ruled out. In Nu4, the proximity to a main highway (450 m westwards) can be also considered a risk factor for airborne transmission. The land surrounding Nu4 is flat land with cultivated fields and no farms are in the vicinity (> 1.5 km).

The study of this production system allowed to see the evolution of the infection by this strain of enhanced virulence. The first case took place in January 2020, before the onset of the present study and resulted in a devastating impact on the affected farm. The productive data suggested that the introduction of the virus resulted in an explosive dissemination of the infection within the farm, with almost all sows probably infected in a short period of time. Although the farm was positive stable and vaccination against PRRSV was implemented, the behavior of the infection resembled a scenario of a fully naïve population stricken by a new agent. It is worth noting that the infection in M2 was associated with an important mortality in sows. In contrast, when farm M7 was infected, some 11 months later, the impact was different: lower abortion rates, lower mortality of sows, but a higher persistence of the disease and more time needed to return to baseline normality (10–11 months for most of the examined parameters). Finally, for Nu4, the infection produced less abortion, less mortality in sows, but even more time was needed to return to normality, with most parameters still altered 39 weeks after the onset of the outbreak. This farm was the only one that was not implementing vaccination before the outbreak, although it had been classified in the PRRSV-positive category long before the outbreak with the new strain. In comparison, data published for PRRSV-2 in the USA indicated a median time to baseline production of 16.5 weeks (range 0–29 weeks) [14]. These figures may somewhat fit the case of M2, except for sow mortality, but are well below of the figures for M7 and Nu4, suggesting that the impact of the PRRSV-1 strain affecting the present production system was probably above the average of PRRSV-2 infected farms in the USA. Time to stability was not established since, at the end of the study, weaned animals of the farms infected by the increased virulence isolate were still testing positive.

Looking at the mean substitution rates of each clade of the virus, it can be observed that while the clade affecting M2 (the first farm infected) showed a substitution rate of 1.1%, the mean substitution rate for M7 was 0.84 and it was 0.33 for Nu4. In other words, later variants of the virus showed less acute impact in the farm, but more persistence, together with lower substitution rates. It is tempting to hypothesize that the virus reduced its virulence as gained capability to persist in the herd, in the framework of a selection process. The more adapted the virus and the more fit for persistence, the lower the substitution rate. Compared to the evolution of the Spanish clades already existing in the production system (mean substitution rates from 0.03 to 0.17%), it is evident that the evolution of the new strain and the already existing ones was taking place at very different speeds, probably reflecting the high dissemination of the new strain. To note, in our hands, the in vitro replication of the highly virulent strain produced titers 1–2 log_10_ higher than those of the classical Spanish PRRSV-1 strains (not shown).

It is also worth to comment that up to four significantly different clades of the emergent PRRSV were detected, suggesting that these were the result of 4 different introduction events from different sources. The sources of the virus could not be established.

## Conclusions

A new highly virulent PRRSV1 strain resulting from different recombination events emerged in Spain in 2020. This strain had a considerable impact in the affected farms, regardless of whether they were previously PRRSV positive and were implementing a vaccination program. However, the impact changed over time, transitioning from a relatively short (3 months), but devasting impact on the reproductive performance, to a milder but sustained impact that required almost one year to return to baseline production. These results highlight the importance of implementing surveillance and reporting systems that may alert of the arising of new PRRSV variants of concern, as well as of the need to extreme the biosecurity of pig farms in Europe.

## Material and methods

### Description of the production system

**Fig. 5:**
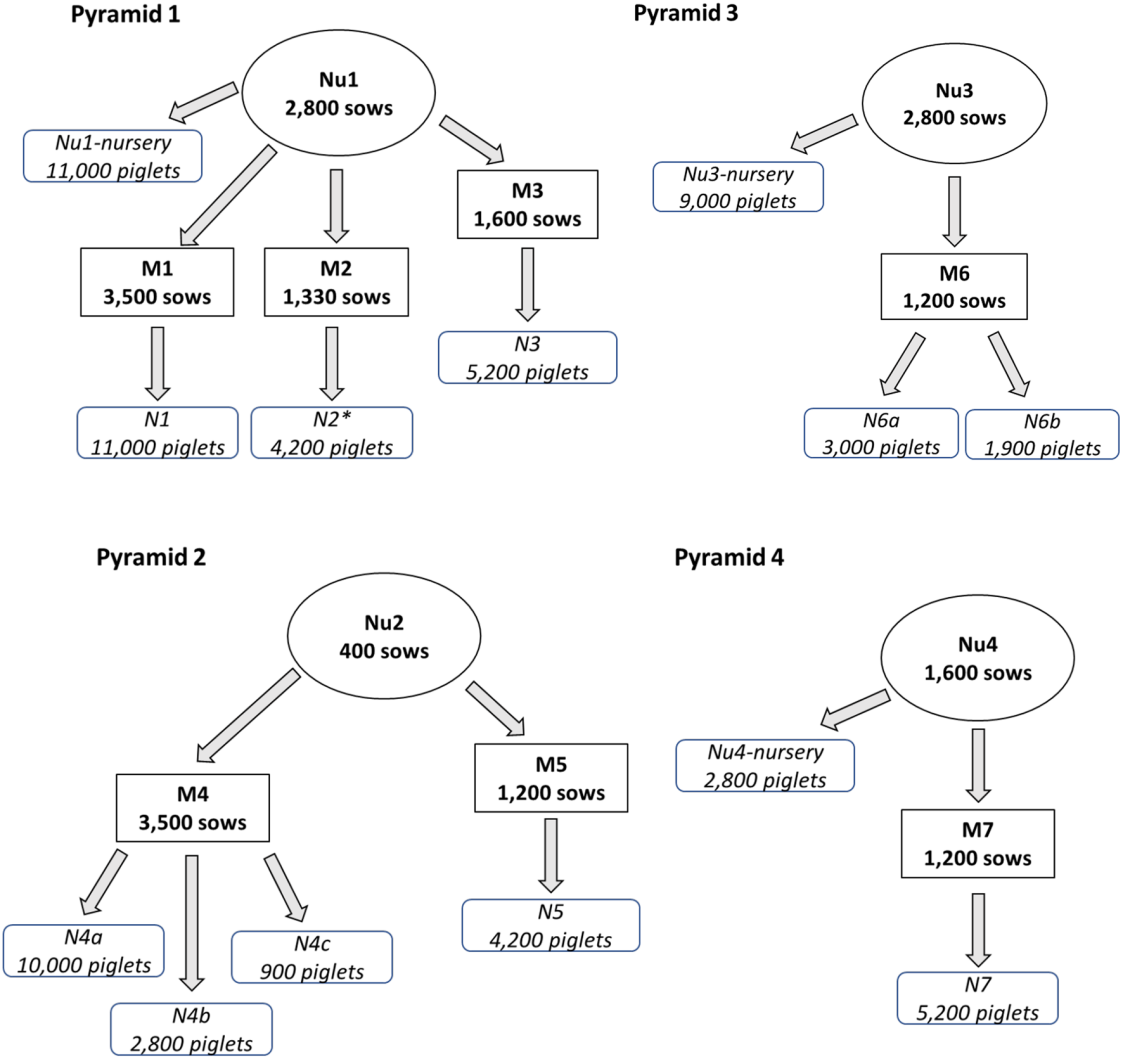
Structure of the production pyramids included in the study

The production structure consisted of four pyramids that accounted for 7,600 sows in 4 nuclei and > 13,000 sows in multipliers (Fig. 5). An additional farrow-to-finish farm (1,600 sows) belonging to the same company sometimes received surplus animals (weaners) from other farms of the system.

All nuclei and breeding farms applied strict biosecurity measures including, but not limited to, double perimetral fence, parking area, limitation of access to farms to non-authorised people, compulsory shower before entering the animal facilities, workwear of exclusive use for each farm, and ≥ 24 h downtime. All trucks used for the transportation of pigs were washed and disinfected after each transport and disinfected again before the transportation of a new batch of animals the next day of operation. Dead animals were disposed in tightly closed containers located outside the perimetral fence. The containers were picked up by trucks designed for this purpose and that never entered the perimeter of the farm.

Farms on top of each pyramid and the farrow-to-finish farm only used self-replacements. In all cases, gilts sent from the nucleus farms (Nu1-Nu4) to the multipliers were tested for PRRSV by RT-qPCR at weaning and only negative gilts were sent to the destination farm. Each pyramid was operated independently and had no contact with the others through personnel, trucks or by any other mean. PRRS-positive farms were vaccinating gilts and sows against PRRSV with modified-live vaccines (twice before first service and recall blanket vaccination every four months with UNISTRAIN PRRS®) except for Nu4 that did not apply vaccination against PRRSV.

Regarding the location of the farms, all were located ≥ 2.5 km form other pig farms except for M2, that was located 800 m from a fattening unit belonging to a different company. Nu4 was located at < 1 km from a highway with high density of pig transports.

Pyramid 1 (P1) included one nucleus (Nu1, 2,800 sows), three multipliers (M1, M2, M3, 6,430 sows in total) and several nurseries that then feed other production farms (within or outside the company) with breeders or fatteners. Nu1 was PRRSV-positive at the beginning of the study (December 2020), as revealed by the occasional detection of positive sows or piglets. M1 was stable to PRRSV (no RT-PCR positive pigs at weaning), but in M2 and M3 sows or piglets were occasionally found to be positive for PRRSV by RT-PCR. Pyramid 2 (P2) consisted of a nucleus (Nu2, 400 sows) that sent sows to two breeding farms (M4, M5; 4,700 sows in total), plus several nurseries that fed several other farms as before. Nu2 and M5 were historically free from PRRSV, while M4 was known to be endemically infected with viral circulation in the farrowing units. Pyramid 3 (P3) included one nucleus (Nu3, 2,800 sows), one multiplier (M6; 1,200 sows) and several nurseries. M6 was known to be endemically infected by PRRSV at the beginning of the study. Pyramid 4 (P4) included one nucleus (Nu4, 1,600 sows), one multiplier (M7, 1,200 sows) and two nurseries that fed other farms outside the pyramid or sent animals for fattening. Nu4 was PRRSV-positive with occasional detection of infected animals at weaning; M7 was PRRSV positive but stable, and circulation of the virus was not detected in weaners. The farrow to-finish farm M8 was endemically infected by PRRSV. After March 2021, M6 sent weaned pigs to nursery N7 that was previously used for weaners coming from M7 (M7 growers were sent to nursery N6a afterwards).

### Sampling and RT-qPCR analysis

In this study (December 2020–October 2021) samples taken for routine PRRSV monitoring or diagnosis were used. All nucleus and breeding farms were monitored monthly for PRRSV. Results included in the present study encompassed the period between December 2020 and October 2021. In breeding herds, 30 random suckling piglets at 3 weeks of age were bled monthly for monitoring. When a reproductive outbreak compatible with PRRSV appeared, sampling was aimed at affected litters and sows. In nurseries, monitoring was performed every month by a cross-sectional sampling at 6 and 9 weeks of age (10 animals each). Samples were submitted to the Veterinary Laboratory for the Diagnosis of Infectious Diseases of UAB for diagnosis. Upon reception, serum was separated, and RNA was extracted using the MagMax Core RNA Extraction kit in a KingFisher Flex System (ThemoFisher). Samples were analyzed by RT-qPCR using the LSI VETMAX PRRSVEUNA 2.0 kit (Thermofisher).

### Impact of the infection by a new highly virulent PRRSV strain in previously PRRSV-infected farms

To assess the impact of the infection caused by the new highly virulent isolate, productive and mortality data were collected from 3 farms (M2, Nu4 and M7) that were already PRRSV-positive because of previous infections and that in two cases (M2 and M7 were vaccinating). Data collected included the numbers of served sows, number of abortions, mortality of productive sows, total number of piglets born per sow (broke down as born alive, mummified, stillbirths), mortality from birth to weaning, and fertility rates. Two nurseries (N2 and N7) were also included in the analysis (mortality rates, feed conversion index from weaning to 10 weeks of age, and average daily weight gain). Analysed data comprised 2019, 2020 and 2021. Average data for each farrowing batch (weekly) were rolling averaged (three weeks). Return to baseline for a given parameter was considered to happen when, for at least 4 consecutive weeks, the values for that parameter were in or above the median of the farm calculated for the six months before the outbreak. Farm M4 and nursery N4 that were endemically infected by a Spanish strain of moderate virulence since before 2019 were used for comparative purposes. M4 applied a vaccination plan to gilts and sows as mentioned above.

### Virus isolation, sequencing, and phylogenetic analyses

Virus isolation in alveolar macrophages was attempted using the samples showing the lowest Cts for PRRSV. To avoid biasing the results, only a single passage was performed. According to previous reports [8], with this method the discrepancy between the PRRSV sequence obtained from the original sample, or from the isolate was 1–3 nucleotides per 10^4^ positions. Viral RNA was extracted from the isolates using the Trizol reagent (Thermo Fisher), with an elution volume of 20 µl.

The presence of PRRSV RNA in the sample was assessed by using a commercial RT-PCR kit (LSI VETMAX PRRSVEUNA, ThermoFisher), according to the instructions provided with the kit. If the Ct of the sample was lower than 20, the viral RNA was used for NGS using Illumina Miseq without performing any previous amplification. The protocol developed in five steps. First, the genomic library was constructed using a commercial protocol and reagents (Protocol for use with Purified mRNA or rRNA Depleted RNA and NEBNext® Ultra™ II RNA Library Prep Kit for Illumina®, New England Biolabs). After the NGS run, sequences of low quality were trimmed (QC > 20) using Trimmomatic [3]. Then, reads were mapped against a reference sequence (Burrows-Wheeler Aligner applying the BWA-MEM algorithm for long reads) [13]. The reference sequence was produced form the earliest available isolate obtained during the outbreak by de novo assembly using SPADES [1]. In the fourth step, variant calling to determine the frequency of each nucleotide at each position of the reference genome was performed with SnpSift [7]. Finally, the viral quasi-species was constructed in fasta format and the consensus sequence was obtained using Consensus software (http://www.hiv.lanl.gov/content/sequence/CONSENSUS/consensus.html).

A phylogenetic tree was built up for the whole genome sequences using MEGA X [24], applying the maximum likelihood method, a general time reversible model with 1,000 iterations and pairwise deletion. The dataset contained, apart from the 55 consensus sequences obtained in this work, two reference PRRSV1 genomes (NC_043487-Lelystad and JF276435-CReSA3267), plus 4 vaccine strains available in GenBank. The tree was rooted with a PRRSV1 subtype 3 strain (JF802085-Lena).

### Recombination analyses

The existence of potential recombination points within the viral genome was initially assessed using GARD Kosakovsky Pond [12] and RDP v5 (Darren et al. 2021). The dataset was the same used for the phylogenetic analysis. For every sequence, the recombinant segments delimited by the recombination points detected by both programs were submitted to BLAST, to identify the potential parental strains.

### Substitution rate estimations

The evolutionary rate within every clade of related transmission -for the whole genome, as well as per every genome segment- were calculated using a Bayesian Markov chain Monte Carlo (MCMC) approach, implemented in BEAST v1.10.4 [23]. Firstly, the aligned consensus sequences of every isolate were used to generate a BEAST input file, using the software BEAUTi. Three independent runs of MCMC per dataset were performed under a strict molecular clock model, using the Tamura-Nei model of sequence evolution, with a proportion of invariant sites and gamma distributed rate heterogeneity (TN + I + Γ), with partitions into codon positions, and the remaining default parameters in the prior’s panel. Every MCMC run was 100 million steps long and the posterior probability distribution of the chains was sampled every 1000 steps. Convergence was assessed based on an effective sampling size after a 10% burn-in, using Tracer v1.7.2 [21]. The substitution rate estimations were the mean values obtained for the three runs.

## Supplementary Information

**Additional file 1**. Emergence of the different clades of the highly virulent PRRSV isolate or its recombinants.

**Additional file 2**. Recombination patterns among the recombinant isolates detected for the new strain of enhanced virulence.

**Additional file 3**. Variation in the number of weaned piglets with regards to the pre-established production objectives of each farm.

**Additional file 4**. Excess mortality in nurseries associated to the PRRS outbreak.

## Data Availability

Sequences are available at Genbank under accession numbers OM893828-OM893841, OM893845-OM893855 and OP822960-OP822989. Productive data can be available from the authors upon reasonable request.
