# Supplementary material for: Introduction of a PRRSV-1 strain of increased virulence in a pig production structure in Spain: virus evolution and impact on production
Source: Porcine Health Manag. 2023 Jan 3;9:1. doi: 10.1186/s40813-022-00298-3 (PMC9811746; doi:10.1186/s40813-022-00298-3)
Supplement: Supplementary file 1 — Additional file 1. Emergence of the different clades of the highly virulent PRRSV isolate or its recombinants. [file 40813_2022_298_MOESM1_ESM.docx]

**Supplementary material 1**

**S1. Emergence of the different clades of the highly virulent PRRSV isolate or its recombinants.** The table shows the date in which the new strain was detected and the related cases.

| **Cluster** | **First detection/farm** | **Related infected farms** |
| --- | --- | --- |
| 1 | January 2020 / M2 | N2 |
| 2 | March 2021/M8 | N4b |
| 3 | March 2021/N7 | N5, N6a, M7 |
| 4 | May 2021/Nu4 | M7 |
