# Supplementary material for: Introduction of a PRRSV-1 strain of increased virulence in a pig production structure in Spain: virus evolution and impact on production
Source: Porcine Health Manag. 2023 Jan 3;9:1. doi: 10.1186/s40813-022-00298-3 (PMC9811746; doi:10.1186/s40813-022-00298-3)
Supplement: Supplementary file 2 — Additional file 2. Recombination patterns among the recombinant isolates detected for the new strain of enhanced virulence. [file 40813_2022_298_MOESM2_ESM.pptx]

## Slide 1
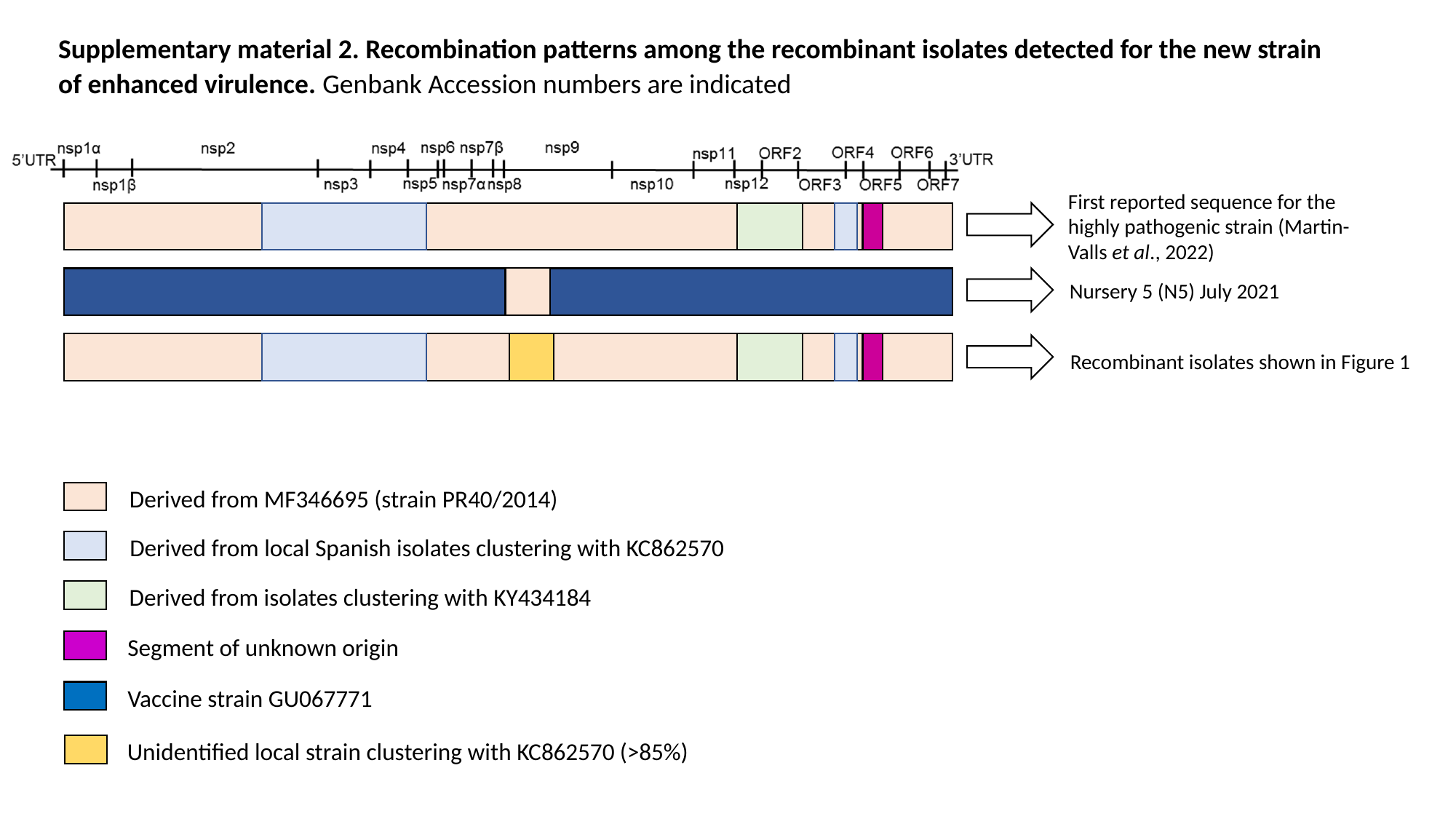

Supplementary material 2. Recombination patterns among the recombinant isolates detected for the new strain of enhanced virulence. Genbank Accession numbers are indicated
First reported sequence for the highly pathogenic strain (Martin-Valls et al., 2022)
Nursery 5 (N5) July 2021
Recombinant isolates shown in Figure 1
Derived from MF346695 (strain PR40/2014)
Derived from local Spanish isolates clustering with KC862570
Derived from isolates clustering with KY434184
Segment of unknown origin
Vaccine strain GU067771
Unidentified local strain clustering with KC862570 (>85%)
