# Supplementary material for: Introduction of a PRRSV-1 strain of increased virulence in a pig production structure in Spain: virus evolution and impact on production
Source: Porcine Health Manag. 2023 Jan 3;9:1. doi: 10.1186/s40813-022-00298-3 (PMC9811746; doi:10.1186/s40813-022-00298-3)
Supplement: Supplementary file 3 — Additional file 3. Variation in the number of weaned piglets with regards to the pre-established production objectives of each farm. [file 40813_2022_298_MOESM3_ESM.pptx]

## Slide 1
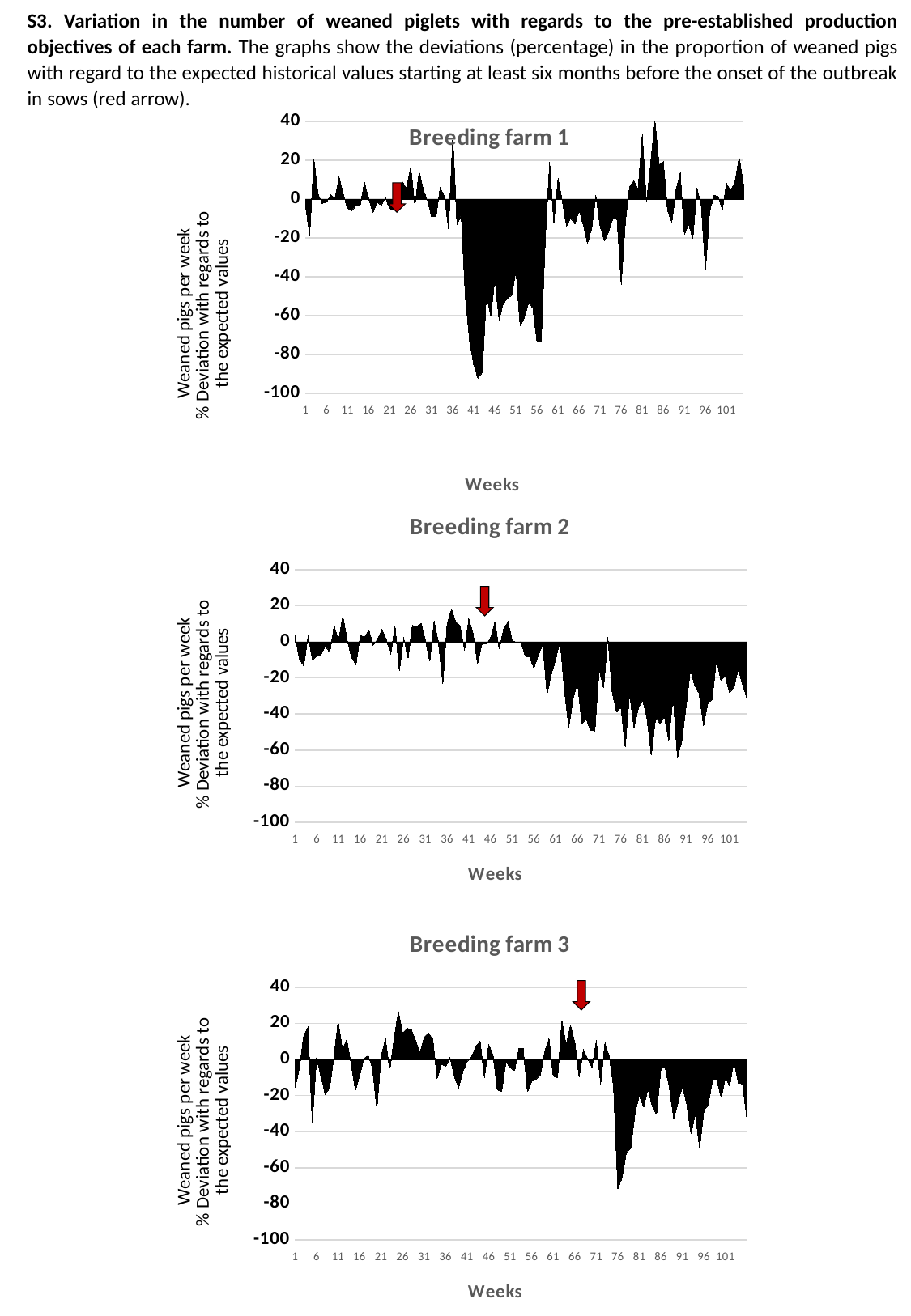

S3. Variation in the number of weaned piglets with regards to the pre-established production objectives of each farm. The graphs show the deviations (percentage) in the proportion of weaned pigs with regard to the expected historical values starting at least six months before the onset of the outbreak in sows (red arrow).
### Chart: Breeding farm 1
| Category | Farm 1 |
|---|---|
| 1 | -2.8783902012248497 |
| 2 | -18.79265091863517 |
| 3 | 20.621172353455812 |
| 4 | 2.773403324584424 |
| 5 | -2.1347331583552256 |
| 6 | -1.5398075240595124 |
| 7 | 2.1784776902887106 |
| 8 | 0.6911636045494284 |
| 9 | 11.548556430446192 |
| 10 | 2.3272090988626393 |
| 11 | -4.663167104111989 |
| 12 | -5.853018372703414 |
| 13 | -3.3245844269466347 |
| 14 | -3.3245844269466347 |
| 15 | 8.72265966754157 |
| 16 | 0.5424321959755001 |
| 17 | -6.894138232720913 |
| 18 | -1.5398075240594955 |
| 19 | -3.0271216097987783 |
| 20 | 0.8398950131233566 |
| 21 | -5.109361329833774 |
| 22 | -5.853018372703414 |
| 23 | -3.175853018372706 |
| 24 | 9.16885389326334 |
| 25 | 5.599300087489061 |
| 26 | 16.605424321959752 |
| 27 | -3.473315835520563 |
| 28 | 14.374453193350828 |
| 29 | 5.004374453193348 |
| 30 | -0.7961504811898543 |
| 31 | -9.125109361329836 |
| 32 | -8.827646544181981 |
| 33 | 6.045494313210845 |
| 34 | 1.4348206474190697 |
| 35 | -15.074365704286965 |
| 36 | 32.519685039370074 |
| 37 | -13.1408573928259 |
| 38 | -8.232720909886266 |
| 39 | -50.91863517060368 |
| 40 | -73.22834645669292 |
| 41 | -85.57305336832896 |
| 42 | -92.26596675415573 |
| 43 | -89.14260717410323 |
| 44 | -48.53893263342082 |
| 45 | -60.13998250218723 |
| 46 | -41.102362204724415 |
| 47 | -62.22222222222222 |
| 48 | -53.59580052493439 |
| 49 | -51.21609798775153 |
| 50 | -49.28258967629047 |
| 51 | -36.491688538932635 |
| 52 | -65.19685039370079 |
| 53 | -61.032370953630796 |
| 54 | -53.00087489063867 |
| 55 | -56.12423447069117 |
| 56 | -73.52580927384076 |
| 57 | -73.37707786526684 |
| 58 | -18.79265091863517 |
| 59 | 18.836395450568673 |
| 60 | -12.397200349956258 |
| 61 | 10.656167979002621 |
| 62 | -0.7961504811898543 |
| 63 | -13.735783027121611 |
| 64 | -9.72003499562555 |
| 65 | -12.843394575678044 |
| 66 | -5.555555555555559 |
| 67 | -13.289588801399827 |
| 68 | -22.659667541557308 |
| 69 | -14.776902887139109 |
| 70 | 1.732283464566926 |
| 71 | -14.033245844269468 |
| 72 | -21.46981627296588 |
| 73 | -16.859142607174103 |
| 74 | -9.868766404199478 |
| 75 | -10.314960629921263 |
| 76 | -43.77952755905512 |
| 77 | -11.058617672790904 |
| 78 | 6.640419947506558 |
| 79 | 9.615048118985124 |
| 80 | 5.153105861767275 |
| 81 | 33.26334208223972 |
| 82 | -1.5398075240594955 |
| 83 | 18.687664041994747 |
| 84 | 40.5511811023622 |
| 85 | 17.944006999125108 |
| 86 | 19.28258967629046 |
| 87 | -5.555555555555559 |
| 88 | -11.951006124234473 |
| 89 | 4.706911636045491 |
| 90 | 13.630796150481187 |
| 91 | -18.197725284339462 |
| 92 | -12.694663167104114 |
| 93 | -20.279965004374457 |
| 94 | 5.599300087489061 |
| 95 | -2.8783902012248497 |
| 96 | -36.491688538932635 |
| 97 | -6.2992125984252 |
| 98 | 2.029746281714783 |
| 99 | 1.4348206474190697 |
| 100 | -5.109361329833774 |
| 101 | 7.979002624671913 |
| 102 | 4.706911636045491 |
| 103 | 8.871391076115483 |
| 104 | 21.81102362204724 |
| 105 | 7.6815398075240555 |
### Chart: Breeding farm 2
| Category | Farm 1 |
|---|---|
| 1 | 3.8295591585153472 |
| 2 | -9.537719415784533 |
| 3 | -13.112689267050778 |
| 4 | 3.6741256867211627 |
| 5 | -10.004019831167087 |
| 6 | -7.827951226048501 |
| 7 | -7.050783867077579 |
| 8 | -2.232346241457854 |
| 9 | -5.807316092724101 |
| 10 | 9.114297199517624 |
| 11 | 0.876323194425839 |
| 12 | 14.399035240519902 |
| 13 | 1.0317566662200237 |
| 14 | -8.44968511322524 |
| 15 | -12.646388851668226 |
| 16 | 3.518692214926978 |
| 17 | 2.8969583277502395 |
| 18 | 6.627361650810672 |
| 19 | -1.7660458260753 |
| 20 | 1.8089240251909469 |
| 21 | 6.93822859439904 |
| 22 | 1.4980570816025778 |
| 23 | -6.895350395283393 |
| 24 | 8.958863727723442 |
| 25 | -15.910491759346105 |
| 26 | 2.275224440573501 |
| 27 | -8.915985528607793 |
| 28 | 9.114297199517624 |
| 29 | 8.647996784135072 |
| 30 | 10.202331502076918 |
| 31 | 1.1871901380142083 |
| 32 | -10.625753718343825 |
| 33 | 11.60123274822458 |
| 34 | -0.056277636339268876 |
| 35 | -23.21586493367278 |
| 36 | 10.357764973871102 |
| 37 | 18.129438563580337 |
| 38 | 11.134932332842027 |
| 39 | 8.958863727723442 |
| 40 | -4.563848318370624 |
| 41 | 12.844700522578057 |
| 42 | 4.606726517486271 |
| 43 | -12.024654964491486 |
| 44 | -1.299745410692746 |
| 45 | -1.299745410692746 |
| 46 | 2.275224440573501 |
| 47 | 11.134932332842027 |
| 48 | -3.9421144311938856 |
| 49 | 7.093662066193225 |
| 50 | 11.290365804636211 |
| 51 | 0.5654562508374698 |
| 52 | -0.2117111081334535 |
| 53 | 0.09915583545491577 |
| 54 | -7.827951226048501 |
| 55 | -8.44968511322524 |
| 56 | -14.511590513198442 |
| 57 | -7.206217338871763 |
| 58 | -1.299745410692746 |
| 59 | -28.81146991826342 |
| 60 | -17.620259949082133 |
| 61 | -9.537719415784533 |
| 62 | 0.7208897226316544 |
| 63 | -26.47996784135066 |
| 64 | -47.3080530617714 |
| 65 | -31.298405466970387 |
| 66 | -21.972397159319303 |
| 67 | -45.59828487203537 |
| 68 | -42.33418196435749 |
| 69 | -49.017821251507435 |
| 70 | -49.32868819509581 |
| 71 | -15.288757872169365 |
| 72 | -25.391933538791367 |
| 73 | 2.4306579123676855 |
| 74 | -28.034302559292506 |
| 75 | -38.914645584885434 |
| 76 | -36.272276564384306 |
| 77 | -58.03296261557015 |
| 78 | -28.656036446469248 |
| 79 | -47.3080530617714 |
| 80 | -36.89401045156103 |
| 81 | -32.23100629773549 |
| 82 | -42.48961543615168 |
| 83 | -62.69596676939568 |
| 84 | -41.71244807718076 |
| 85 | -45.44285140024119 |
| 86 | -41.09071419000402 |
| 87 | -54.613426236098086 |
| 88 | -30.054937692616907 |
| 89 | -63.939434543749165 |
| 90 | -54.92429317968646 |
| 91 | -35.80597614900175 |
| 92 | -15.13332440037518 |
| 93 | -24.303899236232073 |
| 94 | -28.50060297467506 |
| 95 | -46.3754522310063 |
| 96 | -33.78534101567734 |
| 97 | -31.92013935414712 |
| 98 | -9.071419000401978 |
| 99 | -21.35066327214257 |
| 100 | -18.863727723435613 |
| 101 | -28.034302559292517 |
| 102 | -25.081066595202987 |
| 103 | -15.13332440037518 |
| 104 | -23.682165349055335 |
| 105 | -31.14297199517621 |
### Chart: Breeding farm 3
| Category | Farm 1 |
|---|---|
| 1 | -15.583608594695361 |
| 2 | -4.634202178107731 |
| 3 | 13.202734081172117 |
| 4 | 17.97102397226673 |
| 5 | -35.186578146973204 |
| 6 | 1.1937076887856848 |
| 7 | -9.225888739902546 |
| 8 | -19.292278509991174 |
| 9 | -15.936815253294965 |
| 10 | 1.0171043594858844 |
| 11 | 21.14988389966314 |
| 12 | 5.961997579880298 |
| 13 | 11.083494129574513 |
| 14 | -1.9851522386107239 |
| 15 | -16.996435229093766 |
| 16 | -8.872682081302944 |
| 17 | 0.6638977008862833 |
| 18 | 2.253327664584488 |
| 19 | -4.634202178107731 |
| 20 | -27.592634987081798 |
| 21 | 2.253327664584488 |
| 22 | 11.436700788174113 |
| 23 | -6.047028812506135 |
| 24 | 11.613304117473914 |
| 25 | 26.80119043725678 |
| 26 | 14.792164044870324 |
| 27 | 17.264610655067532 |
| 28 | 16.73480066716813 |
| 29 | 10.730287470974911 |
| 30 | 3.842757628282692 |
| 31 | 12.496320763972916 |
| 32 | 14.792164044870324 |
| 33 | 11.260097458874313 |
| 34 | -10.462112045001138 |
| 35 | -2.514962226510126 |
| 36 | -3.751185531608729 |
| 37 | 1.1937076887856848 |
| 38 | -9.402492069202346 |
| 39 | -15.760211923995163 |
| 40 | -6.576838800405536 |
| 41 | -0.9255322628119211 |
| 42 | 1.5469143473852858 |
| 43 | 7.374824214278703 |
| 44 | 10.20047748307551 |
| 45 | -10.108905386401556 |
| 46 | 8.434444190077507 |
| 47 | 2.6065343231840887 |
| 48 | -16.643228570494166 |
| 49 | -17.87945187559278 |
| 50 | -1.1021355921117215 |
| 51 | -4.4575988488079314 |
| 52 | -6.223632141805936 |
| 53 | 5.961997579880298 |
| 54 | 5.961997579880298 |
| 55 | -17.70284854629297 |
| 56 | -12.051542008699352 |
| 57 | -10.815318703600758 |
| 58 | -8.519475422703342 |
| 59 | 4.549170945481895 |
| 60 | 11.613304117473914 |
| 61 | -9.225888739902546 |
| 62 | -9.932302057101747 |
| 63 | 21.50309055826274 |
| 64 | 8.081237531477885 |
| 65 | 19.207247277365337 |
| 66 | 9.67066749517611 |
| 67 | -9.755698727801935 |
| 68 | 5.608790921280697 |
| 69 | 0.48729437158648287 |
| 70 | -4.280995519508131 |
| 71 | 10.377080812375311 |
| 72 | -13.464368643097766 |
| 73 | 9.140857507276708 |
| 74 | 2.0767243352846876 |
| 75 | -15.05379860679595 |
| 76 | -71.74346731203191 |
| 77 | -65.2091441279393 |
| 78 | -51.25748111325507 |
| 79 | -49.314844490957256 |
| 80 | -29.535271609379603 |
| 81 | -19.822088497890576 |
| 82 | -26.356411681983193 |
| 83 | -16.290021911894563 |
| 84 | -26.00320502338359 |
| 85 | -30.241684926578806 |
| 86 | -5.164012166007133 |
| 87 | -4.280995519508131 |
| 88 | -15.936815253294965 |
| 89 | -32.89073486607581 |
| 90 | -23.530758413186383 |
| 91 | -14.70059194819636 |
| 92 | -24.590378388985187 |
| 93 | -41.191091343166434 |
| 94 | -30.065081597279004 |
| 95 | -48.78503450305786 |
| 96 | -28.299048304281012 |
| 97 | -25.120188376884588 |
| 98 | -11.16852536220035 |
| 99 | -11.16852536220035 |
| 100 | -20.88170847368938 |
| 101 | -10.108905386401547 |
| 102 | -14.70059194819636 |
| 103 | -0.39572227491251966 |
| 104 | -13.287765313797955 |
| 105 | -13.464368643097757 |
| | -33.420544853975215 |
