# Supplementary material for: Introduction of a PRRSV-1 strain of increased virulence in a pig production structure in Spain: virus evolution and impact on production
Source: Porcine Health Manag. 2023 Jan 3;9:1. doi: 10.1186/s40813-022-00298-3 (PMC9811746; doi:10.1186/s40813-022-00298-3)
Supplement: Supplementary file 4 — Additional file 4. Excess mortality in nurseries associated to the PRRS outbreak. [file 40813_2022_298_MOESM4_ESM.docx]

Supplementary material 4. Excess mortality in nurseries associated to the PRRS outbreak.

| Farm | Excess mortality in nurseries |
| --- | --- |
| #1 | -373 piglets/batch for 52 weeks  -6.04 piglets/litter |
| #2 | N.D. |
| #3 | -86 piglets/batch for 40 weeks  -1.26 piglets/litter (for 40 weeks) |
